# Discrimination of normal from slow-aging mice by plasma metabolomic and proteomic features

**DOI:** 10.1007/s11357-025-02028-3

**Published:** 2025-12-15

**Authors:** Bretton Badenoch, Oliver Fiehn, Noa Rappaport, Pranjal Srivastava, Kengo Watanabe, Sriram Chandrasekaran, Richard A. Miller

**Affiliations:** 1 Department of Molecular and Cellular Pathology, University of Michigan, Ann Arbor, MI 48103 USA; 2 UC Davis West Coast Metabolomics Center, University of California, Davis, CA 95616 USA; 3 Institute for Systems Biology, Seattle, WA 98109 USA; 4 Phenome Health, Seattle, WA 98109 USA; 5 Buck Institute for Research On Aging, Novato, CA USA; 6 Department of Computational Medicine and Bioinformatics, University of Michigan, Ann Arbor, MI 48109 USA; 7 Department of Medical Artificial Intelligence and Data Science, Graduate School of Biomedical Sciences, Tokushima University, Tokushima, 770-8503 Japan; 8 Department of Biomedical Engineering, Center for Bioinformatics and Computational Medicine, Ann Arbor, MI 48109 USA; 9 Michigan Institute for Data and AI in Society, University of Michigan, Ann Arbor, MI 48109 USA

**Keywords:** Slow-aging mice, Metabolomic, Proteomic

## Abstract

Tests that can predict whether a drug is likely to extend mouse lifespan could speed up the search for anti-aging drugs. We have applied a machine learning algorithm, XGBoost regression, to seek sets of plasma metabolites (*n* = 12,000) and peptides (*n* = 17,000) that can discriminate control mice from mice treated with one of five anti-aging interventions (*n* = 278 mice). When the model is trained on any four of these five interventions, it predicts significantly higher lifespan extension in mice exposed to the intervention which was not included in the training set. Plasma peptide data sets also succeed at this task. Models trained on drug-treated normal mice also discriminate long-lived mutant mice from their respective controls, and models trained on males can discriminate drug-treated from control females. Triglycerides are over-represented among the most influential features in the regression models. Triglycerides with longer fatty acid chains tend to be higher in the slow-aging mice, while triglycerides with shorter fatty acid chains tend to decrease. Plasma metabolite patterns may help to select the most promising anti-aging drugs in mice or in humans and may give new leads into physiological and enzymatic targets relevant to the discovery of new anti-aging drugs.

**Supplementary Information:**

The online version contains supplementary material available at https://doi.org/10.1007/s11357-025-02028-3.

## Introduction

Aging is associated with higher risks of most major contributors to mortality in developed countries, such as cancer, cardiovascular disease, and neurodegeneration [1]. With the aim of postponing aging and age-associated diseases, the National Institute on Aging has for 20 years supported a systematic search for anti-aging drugs [2]. Eleven distinct agents have produced, so far, significant increases in lifespan in one or both sexes, including acarbose (Aca), rapamycin (Rapa), 17-a-estradiol (17aE2), and canagliflozin (Cana) [3–6]. Direct tests of proposed new anti-aging drugs are slow and expensive, however, because lifespan studies in mice require a minimum of 36 months to complete.

Multi-omics analyses, particularly metabolomics and proteomics, have yielded valuable insights into how various anti-aging interventions alter molecular pathways in mice. For instance, partial chemical reprogramming of murine fibroblasts using a small-molecule cocktail significantly enhanced oxidative phosphorylation and reduced aging-related metabolites [7]. In another study, treatment with 17aE2 was found to ameliorate sarcopenia in male mice, evidenced by changes in muscle metabolite levels, chiefly amino acids [8]. Similarly, Aca and Rapa modulated the cardiac and plasma lipidomes of mice [9]. Moreover, proteomic and metabolomic profiling of long-lived growth hormone-releasing hormone knock-out (GHRH-KO) mice emphasized the importance of enhanced mitochondrial function and altered amino acid metabolism in lifespan extension [10]. Collectively, these studies suggest that there are detectable patterns of metabolic change in mice treated with anti-aging compounds, but it is unknown to what degree these interventions overlap.

Our current study makes use of metabolomic and proteomic (peptide-level) data obtained from plasma of young adult (i.e., 12-month-old), genetically heterogeneous [2, 11] mice that had been exposed to an anti-aging drug or to a calorie-restricted (CR) diet from age 4 months. The data set included information on over 12,000 different metabolites and over 17,000 identified peptides. We used a machine learning approach (XGBoost regression) [12]. For each mouse, the percentage lifespan increase was taken to be the median lifespan increase in published data for the intervention in question, and as 0% for untreated control mice. Feature abundance was regressed on this estimated lifespan increase. Estimated lifespan increase for each individual mouse was then calculated based on the abundance of features using the trained regression model. This approach allowed us to elucidate whether metabolomics or proteomics data, or the combination, could serve to make reliable predictions. We also used a “novel intervention test” method, in which the regression model was trained using only four of the five available groups of treated mice, and then used to calculate the estimated lifespan increase for each mouse in the group that was not included in the training procedure, to simulate a situation in which plasma samples were available for mice treated with candidate anti-aging drugs whose effect on lifespan was unknown. We also noted the specific features that contribute most strongly to the performance of each model, with particular attention to changes in the fatty acid constituents of specific triglycerides.

## Methods

### Mouse samples

UM-HET3 mice were weaned at 19–21 days of age and housed at 3 males/cage or 4 females/cage, using the same conditions developed for the Interventions Testing Program [2]. At 4 months of age, mice in the CR group were given 80% of the amount of chow consumed by age- and sex-matched control mice, reduced to 60% at 5 months of age. CR mice were fed once per day, between 8 and 9 am. All other mice had ad libitum access to food. UM-HET3 mice in the other intervention groups began drug treatment at 4 months of age. Mice were not fasted prior to euthanasia. Mice were euthanized at 11–12 months of age, between 8 and 11 am, using CO2 asphyxiation. Mice lost consciousness within 10 s and were removed for closed-chest cardiac puncture as soon as breathing stopped, typically within 30 s. Blood samples were collected using heparin-coated syringes, and plasma removed by centrifugation was then aliquoted and stored at −80 °C until evaluation.

Production of GHRKO and Snell dwarf mice used the breeding schemes described previously [13, 14]. GHRKO mice and their non-mutant littermates were on the C57BL/6 background, and Snell dwarf and their non-mutant littermates were on a segregating stock including 25% C3H/HeJ and 75% DW/J background genes. These mutant mice were 11–12 months of age when euthanized.

All of the animal experiments were approved by the University of Michigan Institutional Animal Care and Use Committee (IACUC). All of the experiments were conducted following the guidelines of this IACUC committee and the University’s Unit for Laboratory Animal Medicine. All of the mice were born and raised at the University of Michigan, using breeding stock purchased from the Jackson Laboratory (Strain #036603).

### Analysis of metabolites in plasma

Plasma metabolites were acquired by five different mass spectrometry techniques: four liquid chromatography-accurate mass spectrometry methods (LC-MS) and one gas chromatography-mass spectrometry assay (GC-MS). Details are given in [15]. Briefly, 20 μl plasma samples was extracted by 1 ml methanol/water/MTBE [16], yielding a lipophilic phase and a hydrophilic phase. Extraction phases were separated, dried, and prepared for LC-MS and GC-MS assays. Data was acquired by hydrophilic interaction chromatography (HILIC)-Orbitrap QE HF mass spectrometry under positive and negative mode electrospray ionization for polar metabolites [17], plus positive and negative mode electrospray ionization for lipids using C18 reversed phase chromatography (RPLC)-Orbitrap QE HF mass spectrometry [18], plus positive mode electron ionization GC-MS using a nominal mass time of flight mass spectrometer (Leco Pegasus IV GC-TOF MS). Data from LC-MS assays was processed in MS-DIAL 4.90 with compound annotations using NIST20 and MassBank.us mass spectral libraries. GC-TOF MS data was processed in ChromaTOF 4.0 software with compound annotations in the BinBase database [19]. Data was normalized using random forest machine learning SERRF software by matching to quality control pool samples [20]. Annotated metabolites (at Metabolomics Society Initiative levels 1–3, MSI [21]) were filtered to have signal/noise ratios *s*/*n* > 3, and unknown metabolites were filtered at *s*/*n* > 10 in comparison to method blank negative controls for local noise estimations.

### Generation of peptides data in plasma

Generation of the peptide data was performed as previously described from the Moritz lab [22].

### Datasets

The datasets used in this paper include plasma metabolomics and plasma peptides. Two versions of the metabolomic dataset were used. The AM data (“all metabolites”) included all the metabolites detected, while the FM (“filtered metabolite”) dataset included only structurally annotated metabolites with MSI = 1, 2, or 3. Similarly, we used two peptide datasets. The AP (“all peptide”) group contained data on each peptide detected, while the FP (“filtered peptide”) dataset was created by retaining only peptides that had no missing data among the set of control mice. All four datasets were Log2-transformed. These datasets were also combined to create the AMP dataset (AM + AP) and FMP dataset (FM + FP). Table 1 shows the number of features within each of these six datasets.

**Table 1:** Six data sets used

Dataset	Number of features	Number of male mice
AM	12,320	148
FM	1051	148
AP	17,628	151
FP	6651	151
AMP	29,949	127
FMP	7701	127

The number of male mice (including control and treated groups) that contributed data to these six sets is shown in the table. All of the data for training were derived from UM-HET3 mice at 12 months of age. The rationale for focusing on models trained on data from males is presented in the text

### Lifespan estimation for groups of treated mice

We utilized a machine learning approach, XGBoost regression [12], to estimate the median percentage increase in lifespan associated with various treatments in mice, independently for each of the six available datasets shown in Table 1. XGBoost was selected due to its ability to handle large, complex datasets with high dimensionality and its effectiveness in identifying non-linear relationships between features and outcomes. For each feature (peptide or metabolite abundance), the dependent variable, median percentage increase in lifespan, was provided based on values in the published literature. Table 2 shows, for each intervention, the published percent lifespan increase and the reference from which the increase was taken. The data are all based on studies of male UM-HET3 mice. Estimates for CR are from the Jackson Laboratory colony and estimates for the other four treatments are derived from the three ITP laboratories, i.e., pooled from Jackson Laboratory, University of Michigan, and University of Texas Health Science Center at San Antonio.

**Table 2:** Published percent lifespan increases for mice for interventions used in this study

Intervention	Dose	Lifespan percent increase (males, females)	Citation
Rapamycin (Rapa)	14.7 ppm	23, 26	[30]
Acarbose (Aca)	1000 ppm	22, 5	[3]
17α-estradiol (17aE2)	14 ppm	12, 0	[3]
Canagliflozin (Cana)	180 ppm	14, 0	[6]
Caloric restriction (CR)	60%	32, 40	Table 1 of [31]
Growth Hormone Receptor Knockout (GHRKO)	Mutant	18, 24	Table 2 of [14]
Snell Dwarf	Mutant	49, 68	Table 2 of [32]

### XGBoost regression models

Data preprocessing involved excluding non-informative and identifier columns, retaining only numerical features relevant for modeling. We employed the eXtreme Gradient Boosting (XGBoost) algorithm to model the relationship between the biological features and the median lifespan increase [12]. The version information for all packages can be found for each module in the git repository (https://github.com/BrettonB/AgingDrugOmics/tree/main/Package_Info). To assess the model’s predictive performance, we initially implemented a tenfold cross-validation strategy. Each dataset was randomly partitioned into ten equal subsets. In each iteration, one subset was used as the test set while the remaining nine subsets constituted the training set. This process was repeated across multiple iterations with different random seeds to ensure the stability of the results. Predictions from all folds and iterations were aggregated to compute median values for each treatment group within each dataset. Statistical significance between treatment groups and controls was assessed using independent Student’s *t*-tests.

### XGBoost novel intervention test (NIT)

For this set of tests, XGBoost models were trained on the contrast between untreated controls and a “treated” group consisting of four of the five interventions (CR diet and four drugs, as in Table 2). The model was then used to estimate the predicted percent increase in median lifespan for each mouse in the treatment group that had been omitted from the training set. This procedure was then reiterated for each of the five varieties of intervention.

### SHAP plots

SHAP values (SHapley Additive exPlanations) were calculated using the Shap package in Python for a model trained with all interventions. Features were then rank-ordered by the SHAP statistic [23] to develop a list of the features with the greatest influence on the estimated percent lifespan increase. The SHAP scores for each of the high-ranked features were then displayed as a log2-fold change graphic. The fold change was calculated by averaging the values of the mice within one intervention group and then dividing by the age-matched control. Then the log2 of this value was displayed to visualize more easily increases and decreases.

### Statistics for contrasts of treated group to untreated control mice

The Student’s 2-tailed *t*-test was performed comparing the control group measurements to the novel intervention measurements.

### Code availability

All custom scripts and workflows used in this study are publicly available at the following GitHub repository: https://github.com/BrettonB/AgingDrugOmics. The plasma metabolite visualization browser tool is available at https://bretton-badenoch.shinyapps.io/Plasma-Anti-Aging-Mouse-Metabolites/.

### Data availability

The metabolomic and peptide plasma LC-MS datasets analyzed during this study is publicly accessible via Zenodo https://zenodo.org/records/17780311.

## Results

### XGBoost regression models trained on published percent lifespan increase for each treatment group

In initial studies, we compared the ability of several varieties of machine learning (ML) algorithms to distinguish between control mice and mice that had been exposed to anti-aging interventions (“treated” mice). The treated mouse group included mice exposed to either rapamycin (Rapa, 14.7 ppm), acarbose (Aca, 1000 ppm), 17α-estradiol (17aE2, 14 ppm), or canagliflozin (Cana, 180 ppm), or which had been exposed to a calorie restricted (CR) diet, adjusted to 60% of the food consumed by age-matched mice. All interventions were initiated at 4 months of age, and plasma samples were taken from 12-month-old mice. Table 1 shows definitions of the datasets and numbers of mice.

**Fig. 1:**
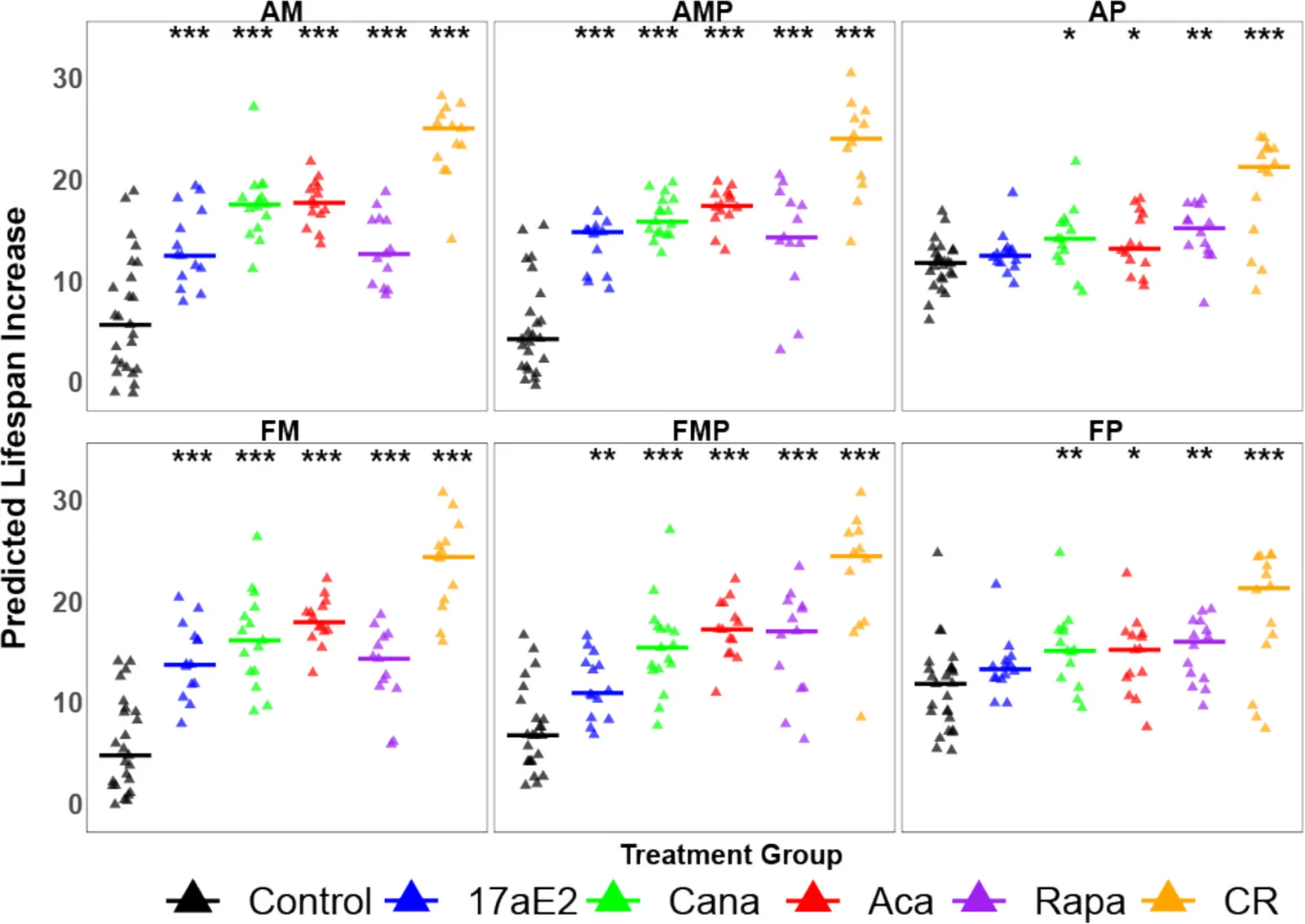
XGBoost regression models including metabolomic features predict percent lifespan increase for male mice for each of five interventions. All data came from analysis of plasma of male mice. Each of the six panels shows results using a different dataset (AM, FM, etc.). Each symbol represents a mouse from the Control group (black symbols) or one of the five indicated intervention groups, evaluated using an XGBoost regression model trained on the indicated data type using tenfold cross validation (see “Methods”). The median value for each intervention group is shown as a horizontal line. Asterisks show significance for *t*-tests contrasting each group of treated mice to control mice, with levels at *p* = 0.05, 0.01, and 0.001, comparing each group of mice to the control mice

**Fig. 2:**
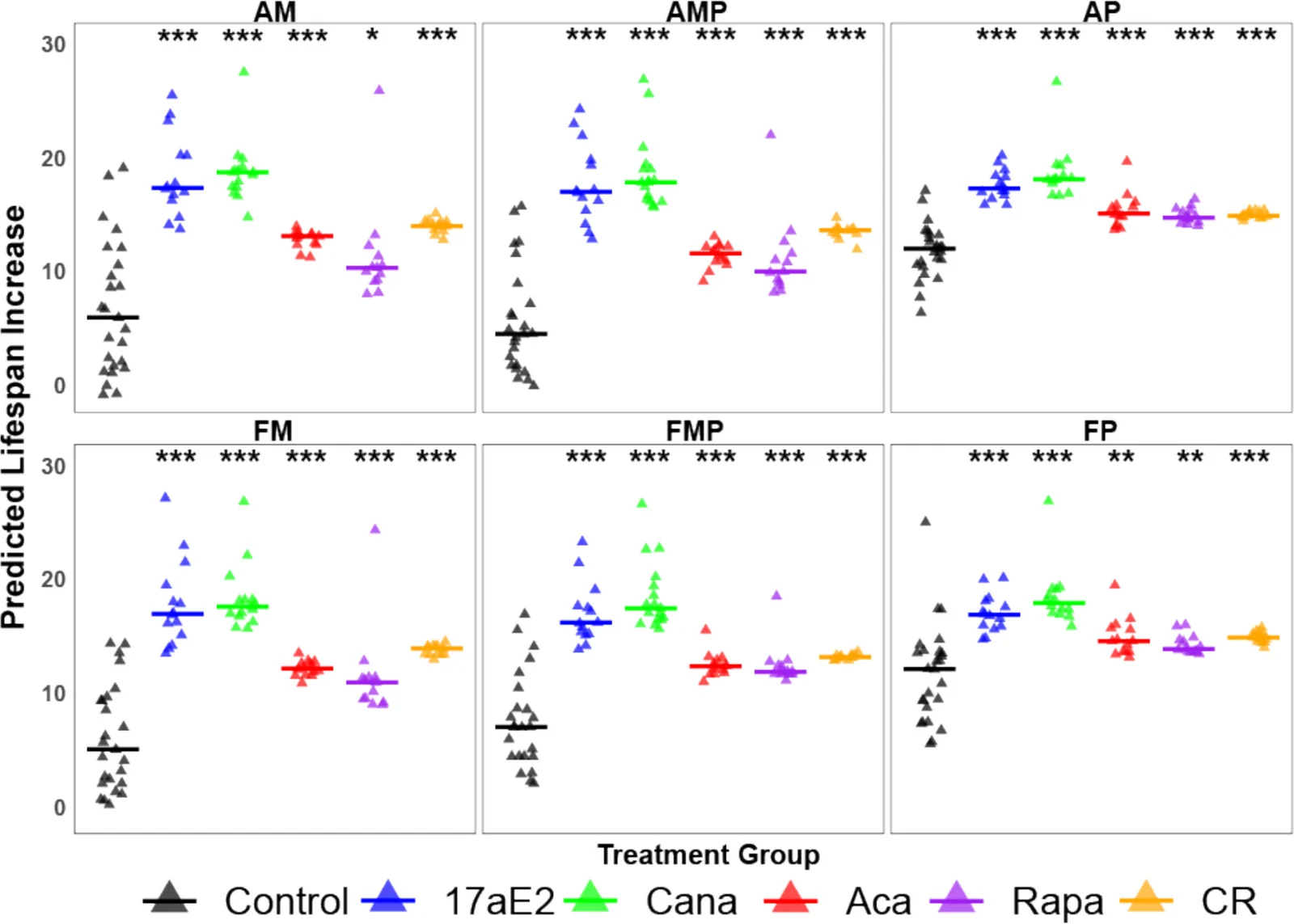
Lifespan estimation for five kinds of slow-aging male mice using XGBoost regression models trained on subsets of four interventions. Each symbol represents one male mouse of the indicated group. For each of the five intervention groups, an XGBoost model trained on controls and the other four treatment groups was used to estimate percent lifespan increase for each mouse in the omitted group (the “novel intervention test.”) For example, symbols for the 17aE2 group reflect a model trained using controls, Cana, CR, Aca, and Rapa mice. Each model was iterated 20 times for each animal, and the median value recorded and plotted in this figure. Horizontal bars show the median value for the group of plotted symbols. Estimated values for control mice were calculated using the same tenfold cross validation method, trained on the entire set of mice (controls and treated), as used for Fig. 1, with 20 iterations for each control mouse. Asterisks show significance for *t*-tests contrasting each group of treated mice to control mice, with levels at *p* = 0.05, 0.01, and 0.001. The specific *p*-values for each group and other summary information can be seen in Supplemental Table 1

**Fig. 3:**
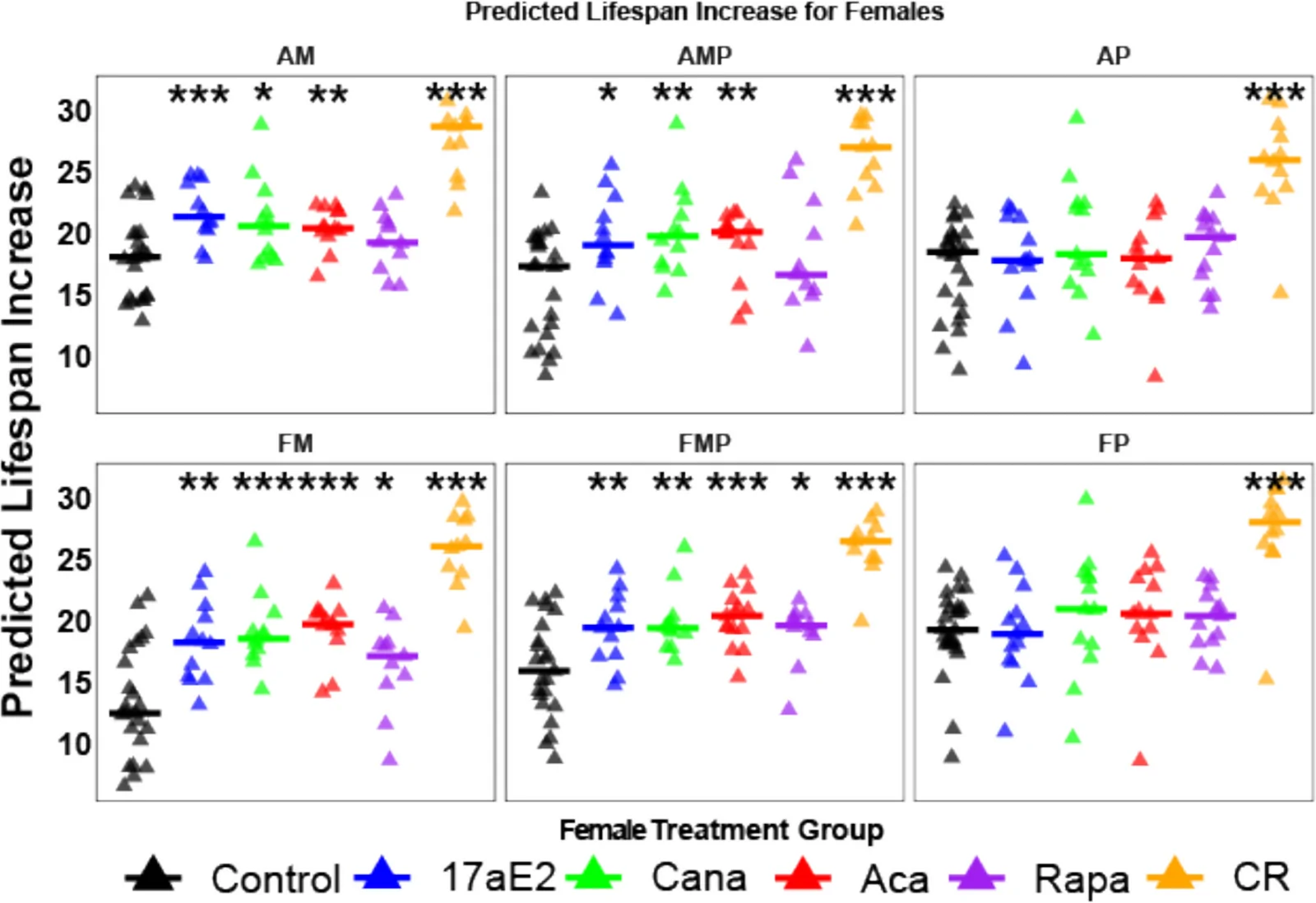
Lifespan estimations for female treated with anti-aging drugs from a male-only trained model. Each symbol represents one female mouse of the indicated group. Each prediction was made once for each mouse. Horizontal bars show the median value for the group of plotted symbols. Asterisks show significance for *t*-tests contrasting each group of treated mice to control mice, with levels at *p* = 0.05, 0.01, and 0.001. 17aE2 and Cana are not known to increase lifespan in females. Acarbose, Rapa, and CR are known to increase lifespan in females

**Fig. 4:**
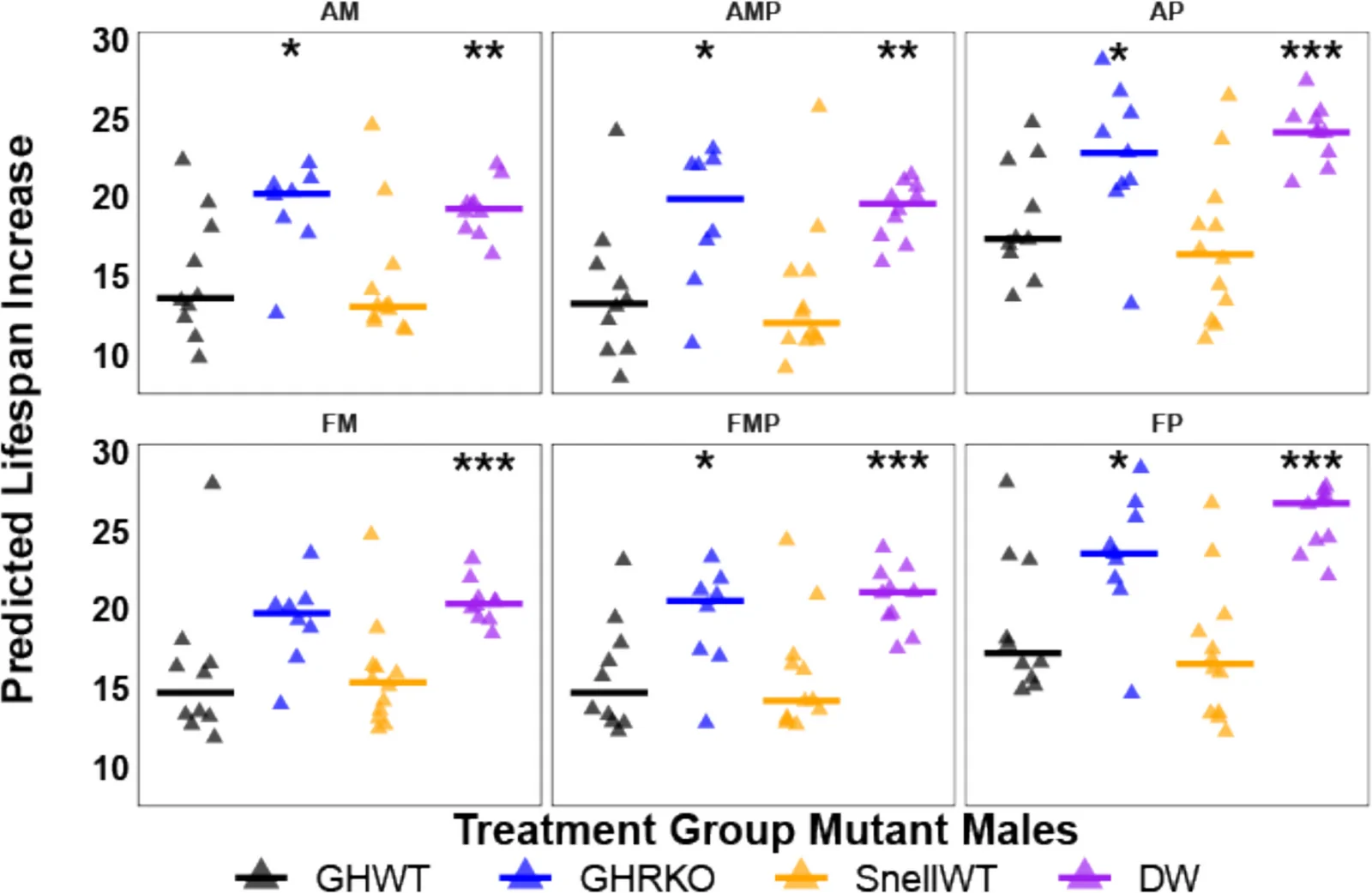
Lifespan estimations for long lived male mutant mice. Each symbol represents one mutant mouse of the indicated group. Each prediction was made once for each mouse. Horizontal bars show the median value for the group of plotted symbols. Asterisks show significance for *t*-tests contrasting each group of treated mice to control mice, with levels at *p* = 0.05, 0.01, and 0.001

The XGBoost regression model was tested for each of the six available data sets (Table 1) using a tenfold cross-validation approach, with results as shown in Fig. 1. Each symbol represents an estimate, for each mouse, of the percent increase in lifespan based on the regression equation. The model was run 10 times for each mouse, each time using 90% of the data and omitting 10%. The median estimate for the series of 10 regressions is then plotted in the figure and used to calculate the mean estimated lifespan value (and standard deviation) for the group. Horizontal lines in Fig. 1 indicate these group-specific mean values, and asterisks indicate the outcome of t-tests comparing groups of treated mice to the group of untreated controls (without adjustment for multiple comparisons). Supplemental Table 1 collects the *p*-values corresponding to each comparison shown in Figs. 1, 2, 3, and 4.

Each of the metabolomic datasets (AM and FM) suggested that this XGBoost regression approach could distinguish control mice from mice in each of the five treatment groups, using *p* < 0.05 as a criterion. The datasets pooling metabolomic and peptide data (AMP, FMP) were at least equally successful in their ability to discriminate between the plasma of control mice and the plasma of mice exposed to an anti-aging intervention. The datasets using peptide data only (AP and FP) did less well by this criterion, suggesting higher estimated lifespan increases only for Aca and CR (and for Cana in the FP data).

### Prediction of estimated lifespan increases for novel treatments

To provide a more realistic and pertinent test of the ability of the XGBoost regression method to characterize novel candidate drugs, i.e., interventions that were not used in the training procedure, we used a “novel intervention test” (NIT) procedure, whose results are shown in Fig. 2. For each mouse in one of the five treatment groups, the model was trained on a dataset consisting of untreated mice and mice in the other four treatment groups but omitting any data from mice in the same treatment group as the tested mouse. This simulates an experimental situation in which the actual lifespan effect of a novel drug is not known and therefore cannot be used in the training set. The estimated lifespan increase of the control mice in Fig. 2 was calculated by 20 iterations of the tenfold cross-validation method used in Fig. 1.

The analysis showed that the XGBoost regression model, applied to the NIT design, was able to correctly indicate an elevated estimate of median lifespan increase for each of the five treatments, even when the model was trained on the other four treatments. It is noteworthy that similar performance was achieved even on the datasets (AP and FP) that did not utilize any of the metabolomic data but relied on peptide data alone.

### Comparison to other ML methods

The results of the XGBoost regression algorithm were compared, via the NIT, to four other ML calculations: support vector regression, K-nearest neighbor regression, HistGradientBoost regression, and random forest regression, using male mice. In each case, we applied the ML method to one of the six datasets. Supplemental Table 2 shows the estimated lifespan increase for each combination of data and method, for each of the five intervention groups, and includes the *p*-values for a comparison of the treated mice to mice in the untreated control group. Only XGBoost regression was able to distinguish each treated group from controls, for each of the six datasets, with a nominal *p* < 0.05. The KNN regression method, for example, did not predict a significant lifespan increase in 8 of the 30 combinations of data and intervention and incorrectly estimated a negative effect of Aca for two of the datasets. This comparison is the basis for our use of XGBoost regression for the remainder of the work shown in this paper.

### Lifespan prediction for female mice using XGBoost regression

Development of ML estimation algorithms for male mice was facilitated by the availability of five different interventions, all of which increased male lifespan by at least 12%. We applied the same approach using models trained on data from female mice and found that these were not able to predict percent lifespan increases for either males or females. We suspect that the models failed because only two interventions, Rapa and CR, led to substantial lifespan increases in female mice, although testing this idea will require the discovery of additional drugs that extend lifespan in females. To see if a model trained on male data would provide useful insights into metabolomic and/or peptide data obtained from female mice, we calculated the predicted percent lifespan increase for female mice in each of the five treatment classes, using the NIT approach with models trained on male data only. The results are shown in Fig. 3 and showed that models trained using metabolomic data from males (AM, FM, AMP, FMP) predicted lifespan increases when applied to plasma from females treated with 17aE2, Cana, or Aca, or exposed to the CR diet. In contrast, females treated with Rapa would not have been correctly identified as long-lived using a model trained on males treated with the other four interventions and control males. Models trained using peptide data only (AP or FP) implied lifespan extension for CR females, consistent with the ability of CR diets to extend lifespan in UM-HET3 females. Male-trained models did not predict lifespan extension in mice treated with Aca, Cana, or 17aE2, consistent with the absence of lifespan extension in female mice exposed to Cana or 17aE2, and with the small (though significant) lifespan extension in Aca-treated female mice. Peptide-trained models did not predict a lifespan effect for Rapa-treated females, although Rapa does indeed extend female lifespan. Thus, models developed using data from plasma of five varieties of slow-aging males show mixed success in predicting lifespan benefits based on plasma data from female mice.

The male-trained models also predicted variable degrees of lifespan extension in untreated female control mice, ranging from 14% to 18%. Female mice of the UM-HET3 stock do typically show lifespans slightly longer than those of male control mice, but the difference is approximately 4% averaged over 13 consecutive annual cohorts.

### Comparison to plasma samples from long-lived mutant mice on two other genetic backgrounds

We also evaluated whether models trained on data from drug- or diet-treated long-lived UM-HET3 mice would reveal similarities to plasma samples from mice carrying mutations that lead to lifespan extension, such as Snell Dwarf (SD) and growth-hormone receptor knockout (GHKRO) mice. The results of these calculations are shown in Fig. 4. Each of the six datasets (metabolites, peptides, or their combination) predicted higher lifespan for SD mice than for their littermate controls. Similarly, five of the six datasets (FM is the exception) predicted significantly higher lifespan in male GHRKO mice than in their own littermate controls. These results suggest that there is some degree of overlap, in both metabolomic and proteomic plasma changes, between long-lived young adult mutant mice and young adult UM-HET3 mice exposed to anti-aging diets or drugs. It also implies that the usefulness of the XGBoost regression model, in the context of the NIT, is not limited to the UM-HET3 stock alone.

### Influential features discriminating control from slow-aging UM-HET3 males

To gain insight into the metabolic features that have the most influence on predicted lifespan change in our UM-HET3 male datasets, we calculated the SHAP value for each metabolite. Table 3 lists the 20 highest-ranked metabolites based on this criterion, and Supplemental Table 3 lists the 100 highest-ranked metabolites in our FM-based models.

**Table 3:** Metabolite name and SHAP value rank

Rank	Feature name	Rank	Feature name
1	N-methylisoleucine	**11**	N-acetyltyrosine
2*	TG 57:9|TG 17:1_18:2_22:6	12*	TG 64:16|TG 20:4_22:6_22:6
3*	TG 48:3|TG 14:0_16:1_18:2	13*	TG O-53:9|TG O-19:5_17:2_17:2
4*	TG 56:9|TG 16:1_18:2_22:6	**14**	N-acetylaspartylglutamic acid
5	N-omega-acetylhistamine	**15**	SM 42:1;2O
6	Ergothioneine	**16**	Sulfamethoxazole
7	1-(piperidin-4-yl)ethan-1-ol	17*	TG 58:8|TG 18:1_18:2_22:5
8	3-hydroxysebacic acid	**18**	N8-acetylspermidine
9*	TG 62:14|TG 18:2_22:6_22:6	**19**	SM 36:1;2O|SM 18:1;2O/18:0
10*	TG 50:3|TG 16:0_16:1_18:2	**20**	1-hexadecanoyl-2-octadecadienoyl-sn-glycero-3-phosphocholine

The metabolites from the FM dataset listed in order of SHAP importance, i.e., influence of the metabolite on predicted percentage lifespan increase. The * indicates that the metabolite is a lipid. TG is an abbreviation for triacylglyceride. Supplemental Table 3 has the mean absolute SHAP values for these 20 features

**Fig. 5:**
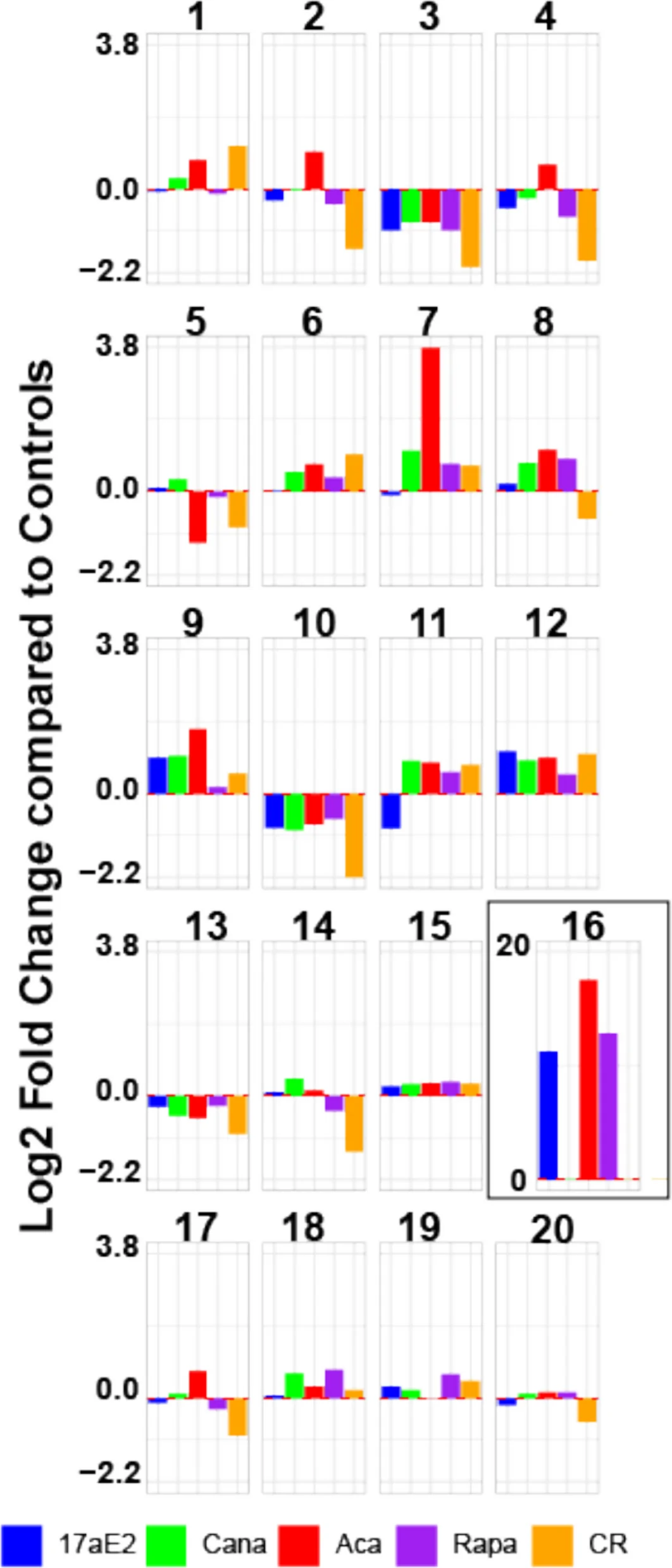
Log2 fold changes of the 20 most informative metabolic features in male mice treated with anti-aging drugs or exposed to the CR diet. Each bar corresponds to the average log2 fold change difference between the treated mice and untreated mice (treated/untreated). The numbers above correspond to the key shown in Table 3. Note altered *Y*-axis scale for Compound 16; this metabolite was undetectably low in plasma samples from all control mice, and the missing values were therefore replaced by the lowest level measured in treated mice

Figure 5 shows the effect of each of the five interventions on each of these 20 features, keyed to the feature number shown in Table 3. Some features (examples: 3, 10) are diminished by all five interventions, with respect to controls. Some features (examples: 9, 12, 18) are increased by all five interventions. There are, however, many features where the direction of the effect is not uniform across all interventions (examples: 2, 4, 5, 8); these features would have been deemed unhelpful using methods that considered only patterns of metabolite change that were uniform in direction across all interventions.

**Fig. 6:**
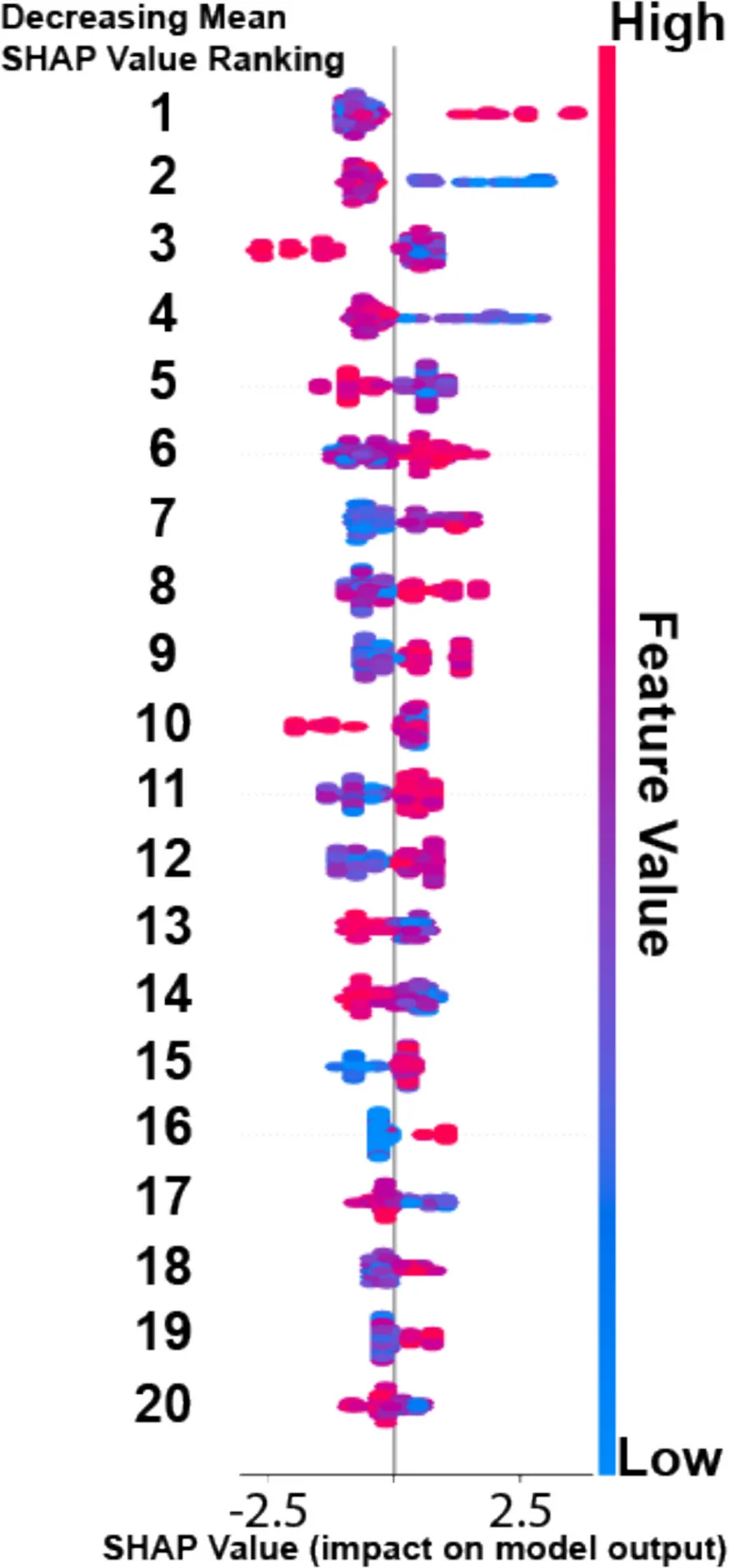
SHAP values for the top 20 predictive features of the model. The color of the SHAP plot corresponds with the magnitude of the feature. Features [1–20] are keyed to the identities listed in Table 3 and shown in Fig. 5. Symbols plotted to the right of the vertical line at *X* = 0 indicate that the value of the feature suggested higher predicted lifespan increase, in an amount proportional to the position of the symbol on the X-axis (SHAP value). Red symbols indicate higher abundance levels, and blue symbols indicate lower abundance

Figure 6 displays the “SHAP plots” for each of these 20 metabolic features, indexed to the features shown in Table 3. Each symbol represents one mouse. Red symbols indicate mice in which high values of the metabolite were influential in the contribution of that metabolite to the overall estimated lifespan outcome. Blue symbols show mice in which low values were influential, and purple symbols show all other mice. Symbols plotted towards the right (values above zero on the *X*-axis) are mice where the metabolite value implied a higher estimated lifespan, and symbols plotted below *X* = 0 contributed to lower estimated lifespan.

**Fig. 7:**
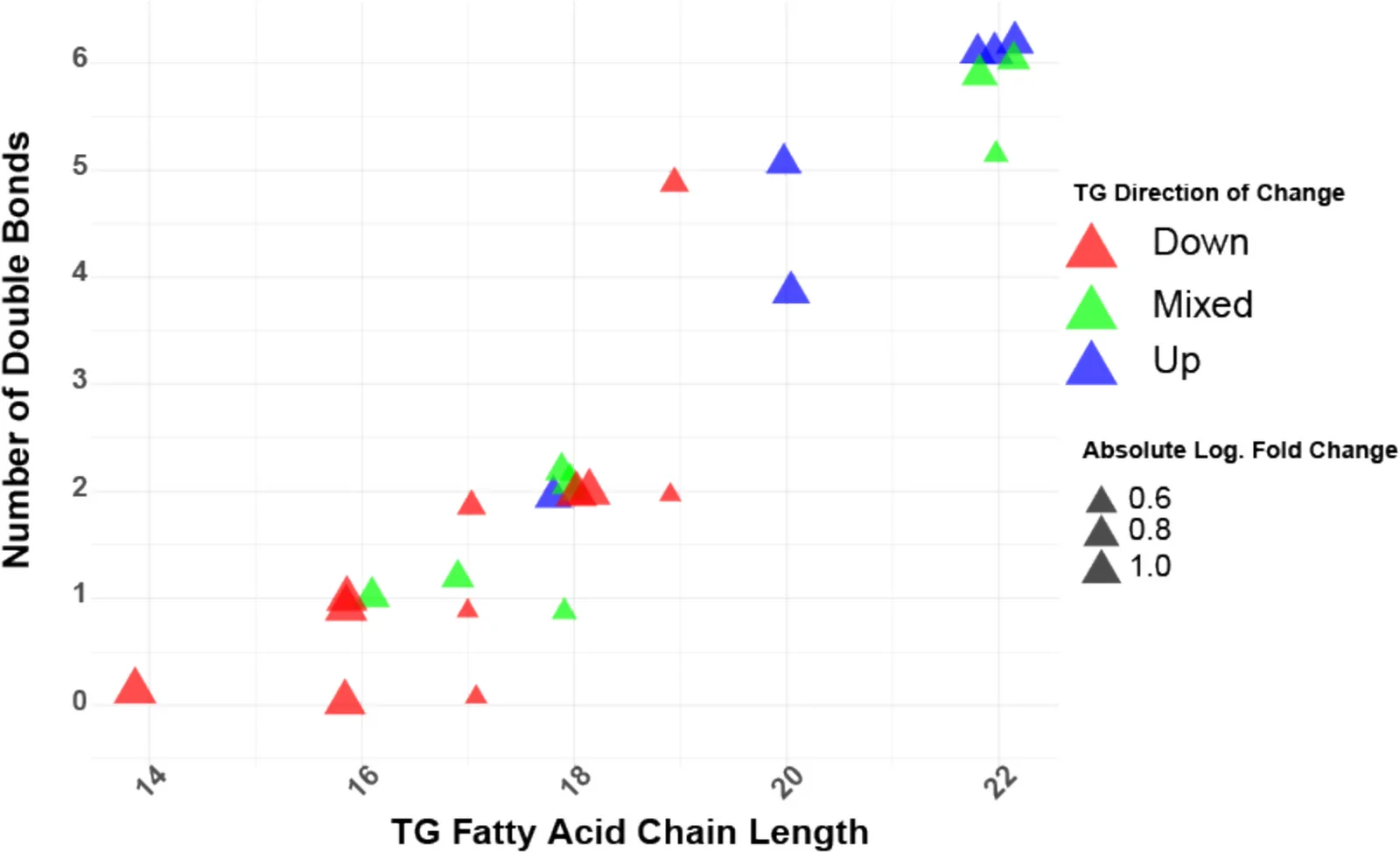
The fatty acid composition of the top 20 most important triacylglycerides in the prediction model. The colors correspond to a change between all treated groups and the untreated control mice. If all the treated groups had lower relative values compared to control mice, then it was colored red; if all the treated groups had higher relative values compared to control mice, then the symbol was colored blue. Green symbols indicate fatty acids which showed a mixture of both increased and decreased levels relative to control mice. The larger the symbol, the larger the magnitude of change in the treated mice compared to the control mice

Of the 20 highest-ranked metabolic features shown in Table 3, 8 are triacylglycerides (TG), suggesting that the anti-aging interventions may often lead to alterations of plasma lipid components. To seek patterns within the TG class, we plotted fatty acid (FA) chain length against double bond number for the 60 FA chains within the 20 highest ranked lipids in the FM data set, using the SHAP score for ranking. Figure 7 shows a clear pattern: with few exceptions, lipids whose FA constituents had longer chain lengths tended to increase in slow-aging mice, while lipids whose FA chain lengths were shorter (18 carbons or less) tended to decrease in the slow-aging mice.

## Discussion

In this study, we developed and evaluated a machine learning model using eXtreme Gradient Boosting (XGBoost) regression to predict the percentage increase in lifespan of male UM-HET3 mice treated with five different anti-aging interventions. Using tenfold cross-validation, in which models are evaluated by successive reservation of 10% of the samples, we found that models that included metabolomic data (AM, FM, AMP, FMP) could successfully differentiate between control mice and mice exposed to any of the five interventions that extend lifespan of males. In the novel intervention test procedure (Fig. 2), each of the six tested datasets, i.e., those based on metabolites or peptides, or on both, is equally successful in discriminating control from treated mice. The implication is that in future applications, when testing candidate drugs whose effects on lifespan are not yet known, data sets containing only metabolite data, or containing only peptide data, can be expected to be just as useful as datasets containing both kinds of data, and the choice of which kind of data to use might be based on relative cost, sample needs, and availability of equipment and expertise.

The “novel intervention test” (NIT) provides a better simulation of a situation in which a candidate drug, not previously tested for lifespan effect, would be evaluated using XGBoost regression trained on data from validated anti-aging drugs and sex-matched controls. In our setting, the model is trained on four of the validated interventions, and estimated percent lifespan increases are calculated for each mouse in the group excluded from the training set. Lifespan estimates are then compared to those of untreated control mice, estimated by the tenfold cross-validation calculation. As shown in Fig. 2, each of the five varieties of treated male mice produced an estimated mean level of lifespan increase that is significantly higher than that of control animals, for datasets comprising metabolites, peptides, or a combination of both kinds of data.

It is noteworthy that all the mice tested in this study were 12 months of age when plasma was taken for testing. This design feature helps minimize effects of aging and age-related diseases on plasma constituents. It also more closely mimics a design in which new candidate drugs could be evaluated by administration to young adults for a comparatively short interval, in the current case 8 months, at less expense and in a shorter time than needed for a complete lifespan study. One goal for future work is to determine if this approach can help prioritize candidate drugs to select a subset that deserves full-scale lifespan testing.

We were unable to develop successful XGBoost models using data from female mice only, and we suspect that this is because only two of the interventions, CR and Rapa, produce lifespan benefits that exceed 7%. We found, however, that the metabolite-trained models developed using FM or FMP data from male mice would have implied extended lifespan in females exposed to any of the five anti-aging interventions (see Fig. 3). This implies that some of the metabolic changes induced by anti-aging interventions in male mice are also produced in female mice, even for drugs (17aE2, Cana) that do not produce significant increases in female lifespan at the doses used. It is possible that these agents might produce a combination of beneficial effects in female mice that are obscured by harmful side effects that limit lifespan [24]. Further exploration of sex effects in this system may have to await the discovery of other interventions that extend the lifespan of female mice.

To see if the metabolic features induced by drugs and diet were also characteristic of mice whose lifespan had been lengthened by a genetic mutation, we looked at plasma from 12-month-old males of two such stocks: Snell dwarf, which are deficient in growth hormone, thyroid stimulating hormone, and prolactin, and GHRKO mice, whose extended longevity is thought to be related to deficient responses to GH alone, without hypothyroidism or low prolactin [14, 25–27]. We found that all six models (trained on metabolites, peptides, or both) predicted elevated lifespans for Snell Dwarf mice, and that five of the datasets made similar correct predictions for lifespan increase in GHRKO mice. The result implies some similarities in metabolic and proteomic effects shared by both mutant and drug/diet-treated mice and that the success of the XGBoost/NIT procedure is not limited to the UM-HET3 genetic stock. Tests of additional long-lived mutants, such as PAPPA-KO and PTEN-transgenic overexpressors, will be needed to explore this issue further.

Of the 20 metabolites that had the greatest influence on the estimation of lifespan predictions (FM dataset), 8/20 (40%) were triacylglycerides (TG), although TG made up only 16% of the metabolites in this dataset (*p* = 0.003). Among the 20 highest-ranked TG (see Supplemental Table 2), FA with 20–22 carbon chains were most often at higher levels in the long-lived mice, while FA with 14–18 carbon chains were typically downregulated. Interestingly, studies in model organisms have demonstrated that manipulation of lipid metabolism can influence lifespan. For example, overexpression of fatty acid desaturase genes, leading to increased production of unsaturated fatty acids like DHA, has been associated with extended lifespan in nematodes [28].

To facilitate the interpretation of the many metabolites in the FM dataset, we developed a freely accessible web tool that displays the relative concentrations of all detected metabolites in both male and female mice from this study, as well as in 6-month-old “Young” UM-HET3 mice. This user-friendly visualization tool enables both researchers and non-experts to explore whether, and how, specific metabolites of interest differ in control versus long-lived mice. Statistical significance (*p* < 0.05), determined by Welch’s two-tailed *t*-test, is indicated by a red asterisk, highlighting differences relative to same-sex controls. The details on where to access the tool can be found in the methods section.

Previous work from this lab [29] has documented shared features seen in each of 10 different varieties of slow aging mice, including the 7 kinds of mice used in the present paper, and argued that these can serve as aging rate indicators (ARIs). Conceptually, ARIs are measures of the instantaneous rate of aging, i.e., the rate at which aging is occurring at the time of sample collection. Unlike biomarkers, ARIs do not require assessment at old age, or at two consecutive ages, since they do not depend on age (or “biological age”), but instead report the rate of age-related change. ARIs shown to date include changes in fat, macrophages, muscle, liver, and brain, as well as two (GPLD1, irisin) proteins present in mouse plasma. We speculate that some of the features shown in Figs. 5 and 6 may prove useful as plasma-based ARIs in future studies and could be put to use in screening of candidate anti-aging drugs in mice, dogs, and/or humans.

The present work has many limitations, which can be addressed by future studies. For one thing, we did not have access to plasma from 12-month-old mice treated with drugs known not to increase UM-HET3 lifespan, and we will need to acquire new samples of this kind to see if the NIT can successfully discriminate drugs known to extend mouse lifespan from those known not to do so. Next, it will be helpful to test the robustness of this approach to data on slow-aging mice generated by other laboratories, in other background stocks, and using other metabolomic pipelines. Application of the approach to metabolic data obtained from dogs of breeds or body weights that differ in expected lifespan will also be of interest. It will be useful to learn if the XGBoost/NIT approach has similar success when applied to internal tissues (liver, brain, kidney, etc.) of slow-aging mice, and, if so, to see to what extent the most influential features in the plasma dataset might overlap with similar lists prepared from various internal tissues. It is also possible that some of these metabolic and proteomic features, in combination, could provide useful insights into changes induced by short-term administration of potential anti-aging drugs in human volunteers.

In summary, our XGBoost regression models suggest that there may be shared metabolic changes produced, at least in male mice, by CR diets and each of four anti-aging drugs with varying biochemical targets and mechanisms. These changes can be seen in 12-month-old (young adult) mice after 8 months of treatment. Some of the changes are also seen in female mice exposed to the same set of treatments as young adults. The alterations can be detected in plasma, which may provide a useful bridge to studies of humans, in which plasma samples are more readily obtainable than samples of internal tissues. Some of the metabolites that have a particularly strong influence on the calculated longevity estimates may be useful as aging rate indicators and help to prioritize new candidate drugs for more intensive evaluation. The list of metabolites with high SHAP scores in our study also implicates specific metabolic and physiological pathways that might help control the aging rate and might usefully be evaluated as targets for the discovery of new anti-aging drugs or nutritional interventions.

## Supplementary Information

Below is the link to the electronic supplementary material.

ESM 1 (DOCX 33.4 KB)
