# Supplementary material for: Discrimination of normal from slow-aging mice by plasma metabolomic and proteomic features
Source: GeroScience. 2025 Dec 15;48(5):7175–90. doi: 10.1007/s11357-025-02028-3 (PMC13601439; doi:10.1007/s11357-025-02028-3)
Supplement: Supplementary file 1 [file 11357_2025_2028_MOESM1_ESM.docx]

**Supplemental Materials**

**Supplemental Table 1: compilation of statistical results for contrasts shown in Figures 1 – 4.**

| **Figure #** | **Dataset** | **Sex_Treatment** | **Pval** |
| --- | --- | --- | --- |
| 1 | AM | M_17aE2 | 8.1E-07 |
| 1 | AM | M_Aca | 2.5E-12 |
| 1 | AM | M_CR | 4.0E-12 |
| 1 | AM | M_Cana | 1.6E-08 |
| 1 | AM | M_Rapa | 6.8E-05 |
| 1 | AMP | M_17aE2 | 9.1E-08 |
| 1 | AMP | M_Aca | 4.3E-12 |
| 1 | AMP | M_CR | 1.2E-11 |
| 1 | AMP | M_Cana | 2.0E-10 |
| 1 | AMP | M_Rapa | 2.1E-06 |
| 1 | AP | M_17aE2 | 1.9E-01 |
| 1 | AP | M_Aca | 3.9E-02 |
| 1 | AP | M_CR | 4.1E-05 |
| 1 | AP | M_Cana | 1.6E-03 |
| 1 | AP | M_Rapa | 9.5E-04 |
| 1 | FM | M_17aE2 | 2.7E-06 |
| 1 | FM | M_Aca | 3.0E-13 |
| 1 | FM | M_CR | 1.2E-11 |
| 1 | FM | M_Cana | 5.5E-08 |
| 1 | FM | M_Rapa | 6.2E-07 |
| 1 | FMP | M_17aE2 | 3.7E-06 |
| 1 | FMP | M_Aca | 2.1E-13 |
| 1 | FMP | M_CR | 1.0E-08 |
| 1 | FMP | M_Cana | 1.4E-11 |
| 1 | FMP | M_Rapa | 1.6E-07 |
| 1 | FP | M_17aE2 | 8.6E-02 |
| 1 | FP | M_Aca | 3.9E-03 |
| 1 | FP | M_CR | 9.1E-04 |
| 1 | FP | M_Cana | 2.1E-03 |
| 1 | FP | M_Rapa | 2.8E-04 |
| 2 | AM | M_17aE2 | 4.3E-09 |
| 2 | AM | M_Aca | 2.6E-05 |
| 2 | AM | M_CR | 2.0E-06 |
| 2 | AM | M_Cana | 1.6E-10 |
| 2 | AM | M_Rapa | 1.1E-02 |
| 2 | AMP | M_17aE2 | 2.1E-10 |
| 2 | AMP | M_Aca | 4.5E-06 |
| 2 | AMP | M_CR | 2.9E-08 |
| 2 | AMP | M_Cana | 7.3E-13 |
| 2 | AMP | M_Rapa | 4.4E-04 |
| 2 | AP | M_17aE2 | 3.7E-12 |
| 2 | AP | M_Aca | 1.6E-06 |
| 2 | AP | M_CR | 3.4E-07 |
| 2 | AP | M_Cana | 5.9E-09 |
| 2 | AP | M_Rapa | 5.9E-07 |
| 2 | FM | M_17aE2 | 9.9E-10 |
| 2 | FM | M_Aca | 6.8E-07 |
| 2 | FM | M_CR | 1.2E-08 |
| 2 | FM | M_Cana | 1.2E-12 |
| 2 | FM | M_Rapa | 7.8E-04 |
| 2 | FMP | M_17aE2 | 5.2E-10 |
| 2 | FMP | M_Aca | 8.3E-06 |
| 2 | FMP | M_CR | 1.1E-06 |
| 2 | FMP | M_Cana | 5.3E-12 |
| 2 | FMP | M_Rapa | 2.2E-05 |
| 2 | FP | M_17aE2 | 2.4E-06 |
| 2 | FP | M_Aca | 1.7E-03 |
| 2 | FP | M_CR | 8.3E-04 |
| 2 | FP | M_Cana | 3.6E-07 |
| 2 | FP | M_Rapa | 6.7E-03 |
| 3 | AM | F_17aE2 | 6.2E-04 |
| 3 | AM | F_Aca | 5.5E-03 |
| 3 | AM | F_CR | 1.0E-08 |
| 3 | AM | F_Cana | 2.4E-02 |
| 3 | AM | F_Rapa | 1.8E-01 |
| 3 | AMP | F_17aE2 | 1.3E-02 |
| 3 | AMP | F_Aca | 6.1E-03 |
| 3 | AMP | F_CR | 6.9E-09 |
| 3 | AMP | F_Cana | 4.5E-03 |
| 3 | AMP | F_Rapa | 2.1E-01 |
| 3 | AP | F_17aE2 | 7.0E-01 |
| 3 | AP | F_Aca | 8.5E-01 |
| 3 | AP | F_CR | 1.2E-05 |
| 3 | AP | F_Cana | 1.2E-01 |
| 3 | AP | F_Rapa | 1.8E-01 |
| 3 | FM | F_17aE2 | 1.2E-03 |
| 3 | FM | F_Aca | 1.1E-05 |
| 3 | FM | F_CR | 2.7E-10 |
| 3 | FM | F_Cana | 1.2E-04 |
| 3 | FM | F_Rapa | 5.2E-02 |
| 3 | FMP | F_17aE2 | 1.0E-02 |
| 3 | FMP | F_Aca | 2.7E-04 |
| 3 | FMP | F_CR | 6.3E-10 |
| 3 | FMP | F_Cana | 1.7E-03 |
| 3 | FMP | F_Rapa | 1.5E-02 |
| 3 | FP | F_17aE2 | 8.8E-01 |
| 3 | FP | F_Aca | 3.8E-01 |
| 3 | FP | F_CR | 2.1E-05 |
| 3 | FP | F_Cana | 3.6E-01 |
| 3 | FP | F_Rapa | 3.2E-01 |
| 4 | AM | M_DW | 2.1E-03 |
| 4 | AM | M_GHRKO | 2.0E-02 |
| 4 | AMP | M_DW | 1.8E-03 |
| 4 | AMP | M_GHRKO | 3.8E-02 |
| 4 | AP | M_DW | 2.3E-04 |
| 4 | AP | M_GHRKO | 5.2E-02 |
| 4 | FM | M_DW | 5.0E-04 |
| 4 | FM | M_GHRKO | 8.8E-02 |
| 4 | FMP | M_DW | 7.4E-04 |
| 4 | FMP | M_GHRKO | 4.6E-02 |
| 4 | FP | M_DW | 2.0E-05 |
| 4 | FP | M_GHRKO | 3.7E-02 |

**Supplemental Table 2: comparison of ML regression models**

**Legend for Supplemental Table 2:** Each pair of columns presents the results of the Novel Intervention Test applied to data from male mice using the ML regression approach listed in the column header. “Lifespan” values are estimated percentage increase in median lifespan averaged over each mouse in the control or tested group. The “P Value” columns give -log10(p) for the t-test comparison of mice in the indicated treatment group to Control mice. Estimates of Control mouse lifespan are based on the 10-fold cross-validation method illustrated in Figure 1. Red highlights indicate p-values that do not reach the p = 0.05 significance criteria, or lifespan increases that are below zero.

**Supplemental Table 3.** The top 100 ranked SHAP scores after 100 iterations of the XGBoost regression algorithm using the FM dataset.

| **Relative impact on model performance** | **Feature** |
| --- | --- |
| 1 | N-Methylisoleucine |
| 2 | TG 57:9\|TG 17:1_18:2_22:6 |
| 3 | TG 48:3\|TG 14:0_16:1_18:2 |
| 4 | TG 56:9\|TG 16:1_18:2_22:6 |
| 5 | N-omega-Acetylhistamine |
| 6 | Ergothioneine |
| 7 | 1-(Piperidin-4-yl)ethan-1-ol |
| 8 | 3-Hydroxysebacic acid |
| 9 | TG 62:14\|TG 18:2_22:6_22:6 |
| 10 | TG 50:3\|TG 16:0_16:1_18:2 |
| 11 | N-Acetyltyrosine |
| 12 | TG 64:16\|TG 20:4_22:6_22:6 |
| 13 | TG O-53:9\|TG O-19:5_17:2_17:2 |
| 14 | N-Acetylaspartylglutamic acid |
| 15 | SM 42:1;2O |
| 16 | Sulfamethoxazole |
| 17 | TG 58:8\|TG 18:1_18:2_22:5 |
| 18 | N8-Acetylspermidine |
| 19 | SM 36:1;2O\|SM 18:1;2O/18:0 |
| 20 | 1-Hexadecanoyl-2-octadecadienoyl-sn-glycero-3-phosphocholine |
| 21 | TG 64:17\|TG 20:5_22:6_22:6 |
| 22 | 1,4-Cyclohexanedicarboxylic acid |
| 23 | 3-Hydroxybutyric acid |
| 24 | TG 53:3\|TG 17:0_17:1_19:2 |
| 25 | N-Methylalanine |
| 26 | PE P-42:6\|PE P-20:0_22:6 |
| 27 | 12R-Hydroxy-5Z,8Z,10E,14Z-eicosatetraenoic acid |
| 28 | TG 46:0\|TG 14:0_15:0_17:0 |
| 29 | N-alpha-Acetyl-arginine |
| 30 | Taurocholic acid |
| 31 | (3-Oxo-2,3-dihydro-4H-1,4-benzoxazin-4-yl)acetic acid |
| 32 | O2_PE 36:1 |
| 33 | Ethyl sulfate |
| 34 | CAR 18:0 |
| 35 | 3-Pyridinemthanol |
| 36 | PC 38:3\|PC 18:0_20:3 |
| 37 | 5-Oxo-1-propyl-2-pyrrolidineacetic acid |
| 38 | PC 42:7 |
| 39 | 3-Hydroxyvaleric acid |
| 40 | 1-(2-Hydroxyethyl)-2,2,6,6-tetramethyl-4-piperidinol |
| 41 | Val-Gln |
| 42 | beta-Hydroxyisovaleric acid |
| 43 | N-Acetyllysine |
| 44 | PC 37:6\|PC 15:0_22:6 |
| 45 | PC 38:5\|PC 18:1_20:4 |
| 46 | TG O-57:1\|TG O-19:1_16:0_22:0 |
| 47 | HexCer 40:1;2O\|HexCer 18:1;2O/22:0 |
| 48 | DG 38:7 |
| 49 | PC 41:6 |
| 50 | TG 55:6\|TG 16:0_18:1_21:5 |
| 51 | Actrarit |
| 52 | 3-Oxo-1,8-octanedicarboxylic acid |
| 53 | TG 46:2\|TG 12:0_16:0_18:2 |
| 54 | TG 64:13\|TG 18:1_22:6_24:6 |
| 55 | DG 50:1 |
| 56 | Ala-Thr |
| 57 | 5-Hydroxy-3-indoleacetic acid |
| 58 | Caffeic acid |
| 59 | 2-Methylglutamic acid |
| 60 | Cholesterol 3-sulfate |
| 61 | Gly-Pro |
| 62 | Epigallocatechin |
| 63 | (3-Carboxypropyl)trimethylammonium |
| 64 | Guanidinopropionic acid |
| 65 | Cytosine |
| 66 | 3-Oxostearic acid |
| 67 | FA 18:3;O |
| 68 | 4-Imidazoleacetic acid |
| 69 | FA 15:0 |
| 70 | 5-Butyl-1H-pyrazole-3-carboxylic acid |
| 71 | Dihydrocapsaicin |
| 72 | PI 36:1\|PI 18:0_18:1_b |
| 73 | 3-Oxocholic acid |
| 74 | 1-Acetylimidazole |
| 75 | Erythronolactone |
| 76 | Glycerophosphocholine |
| 77 | Butyrylcholine |
| 78 | SM 35:2;3O |
| 79 | Cer 41:1;2O\|Cer 18:1;2O/23:0 |
| 80 | PC O-42:7 |
| 81 | 2-Hydroxyphenylacetic acid |
| 82 | 10-Hydroxydecanoic acid |
| 83 | (R)-Butyrylcarnitine |
| 84 | FA 19:0 |
| 85 | 2-Piperidinecarboxamide |
| 86 | TG 46:2\|TG 14:0_16:1_16:1 |
| 87 | 8-Hydroxyquinoline-2-carbaldehyde |
| 88 | TG 62:13\|TG 18:1_22:6_22:6 |
| 89 | CAR 6:0 |
| 90 | 2-Deoxyribose |
| 91 | (4-Methylphenyl)oxidanesulfonic acid |
| 92 | PC O-40:8 |
| 93 | PE 34:1\|PE 16:0_18:1 |
| 94 | Pipecolic acid |
| 95 | 3-Hydroxybutyrylcarnitine |
| 96 | 2-Hydroxyisobutyric acid |
| 97 | SE 28:1/20:3 |
| 98 | (2E)-2-(Propan-2-yl)but-2-enedioic acid |
| 99 | 5-Hydroxy-3,4-dihydro-2(1H)-quinolinone |
| 100 | SM 33:1;2O |
